# Sphingosine-1-Phosphate as a Regulator of Hypoxia-Induced Factor-1α in Thyroid Follicular Carcinoma Cells

**DOI:** 10.1371/journal.pone.0066189

**Published:** 2013-06-18

**Authors:** Veronica Kalhori, Kati Kemppainen, Muhammad Yasir Asghar, Nina Bergelin, Panu Jaakkola, Kid Törnquist

**Affiliations:** 1 Department of Biosciences, Åbo Akademi University, Turku, Finland; 2 Minerva Foundation Institute, Helsinki, Finland; 3 Turku Centre for Biotechnology, Turku, Finland; UAE University, Faculty of Medicine & Health Sciences, United Arab Emirates

## Abstract

Sphingosine-1-phosphate (S1P) is a bioactive lipid, which regulates several cancer-related processes including migration and angiogenesis. We have previously shown S1P to induce migration of follicular ML-1 thyroid cancer cells. Hypoxia-induced factor-1 (HIF-1) is an oxygen-sensitive transcription factor, which adapts cells to hypoxic conditions through increased survival, motility and angiogenesis. Due to these properties and its increased expression in response to intratumoral hypoxia, HIF-1 is considered a significant regulator of tumor biology. We found S1P to increase expression of the regulatory HIF-1α subunit in normoxic ML-1 cells. S1P also increased HIF-1 activity and expression of HIF-1 target genes. Importantly, inhibition or knockdown of HIF-1α attenuated the S1P-induced migration of ML-1 cells. S1P-induced HIF-1α expression was mediated by S1P receptor 3 (S1P_3_), G_i_ proteins and their downstream effectors MEK, PI3K, mTOR and PKCβI. Half-life measurements with cycloheximide indicated that S1P treatment stabilized the HIF-1α protein. On the other hand, S1P activated translational regulators eIF-4E and p70S6K, which are known to control HIF-1α synthesis. In conclusion, we have identified S1P as a non-hypoxic regulator of HIF-1 activity in thyroid cancer cells, studied the signaling involved in S1P-induced HIF-1α expression and shown S1P-induced migration to be mediated by HIF-1.

## Introduction

The bioactive sphingolipid sphingosine-1-phosphate (S1P) has emerged as a potent signaling molecule. It regulates cellular survival, proliferation and motility as well as angiogenesis and inflammation, all processes relevant for tumorigenesis and cancer progression. S1P is normally present in blood at high levels and functions both intra- and extracellularly [1], [2]. Extracellular S1P activates five high affinity S1P receptors (S1P_1–5_) which couple to various G proteins and have both overlapping and opposing effects [3], [4]. Recently, the first intracellular targets of S1P were identified [5], [6]. S1P is produced from sphingosine by sphingosine kinases 1 and 2 (SK1/2). SK1 is considered oncogenic and its expression is elevated in several types of cancers [2].

Hypoxia is a common feature of tumors and the oxygen-sensitive transcription factor Hypoxia-induced factor-1 (HIF-1) a major mediator of cancer progression. HIF-1 target genes help cells adapt to low oxygen levels by regulating glucose metabolism, angiogenesis, survival and invasion. HIF-1 is formed of the oxygen-sensitive regulatory subunit HIF-1α and the constitutively expressed HIF-1β [7], [8]. Under normoxic conditions HIF-1α becomes prolyl hydroxylated, ubiquitylated by the von Hippel Lindau (pVHL) E3 ligase complex and degraded in proteasomes. In hypoxia, prolyl hydroxylase activity is attenuated and HIF-1α protein stabilized [9], [10], [11]. Hypoxia-induced HIF-1α stability also involves the Akt/glycogen synthase kinase 3 (GSK3) pathway which has been shown to act downstream of sphingosine kinase 1 [12], [13]. Additionally, HIF-1α stability in normoxia is regulated by competitive binding of receptor of activated protein kinase C 1 (RACK1) and heat-shock protein 90 (Hsp90). Binding of RACK1 to HIF-1α induces ubiquitylation and degradation while binding of Hsp90 prevents it [14]. HIF-1α translation is regulated by the extracellular signal-regulated kinase (ERK1/2) and phosphoinositide 3-kinase (PI3K)/Akt pathways and their downstream effectors eukaryotic initiation factor 4E (eIF-4E) and p70S6 kinase (p70S6K) [7], [15].

A physiological concentration of S1P strongly increases migration of the ML-1 follicular thyroid cancer cell line [16], an effect which may have contributed to metastasis of the original tumor. We have also shown S1P and vascular endothelial growth factor (VEGF) signaling to cross-communicate in many ways in ML-1 cells. For example, S1P treatment increases both vascular endothelial growth factor receptor 2 (VEGFR-2) expression and VEGF-A secretion while inhibition of VEGFR-2 attenuates several S1P-induced effects [17], [18]. Since S1P and HIF-1 have many similar functions, we investigated whether extracellular S1P is able to affect HIF-1α expression in ML-1 cells. Interestingly, we were able to induce HIF-1α expression in normoxia with pro-migratory, physiological S1P concentrations. This finding led to several questions: does S1P also increase HIF-1 acivity, does S1P-induced HIF-1α mediate S1P-induced migration, what are the signaling pathways involved and what is the mechanism of HIF-1α up-regulation.

In the present study we identify S1P as a non-hypoxic inducer of HIF-1α expression in thyroid cancer cells. S1P increases HIF-1 activity and HIF-1 is involved in S1P-induced migration. Additionally, we show that S1P regulates HIF-1α protein level through a signaling pathway including S1P_3_, G_i_, PI3K, mammalian target of rapamycin (mTOR), MAP kinase kinase (MEK) and protein kinase C βI (PKCβI). We suggest S1P to regulate HIF-1α stability by a pVHL-independent mechanism and HIF-1α synthesis through activation of translational regulators eIF-4E and p70S6K.

## Materials and Methods

DMEM, fatty acid-free BSA, BSA, pertussis toxin (Ptx), cycloheximide (Chx), N-TER Nanoparticle siRNA Transfection System and phorbol 12-myristate 13-acetate (PMA) were from Sigma (St. Louis, MO, USA). FBS, penicillin/streptomycin, L-glutamine, SuperScript III Reverse Transcriptase, First Strand Buffer and RiboGreen RNA Quantitation Reagent were from Invitrogen (Carlsbad, CA, USA). Cell culture plastic ware and human type IV collagen were from Becton Dickinson Biosciences (Bedford, MA, USA). Transwell Permeable Supports were from Corning Inc. (Corning, NY, USA). D-*erythro*-sphingosine-1-phosphate (S1P), SEW-2871, wortmannin, 17-(allylamino)-17-desmethoxygeldanamycin (17-AAG) and antibody for Hsc70 were from Enzo Life Sciences (Plymouth, PA, USA). VPC-23019 was from Avanti Polar Lipids (Alabaster, AL, USA). HIF-1 inhibitor, p70S6K inhibitor, U0126 and JNJ-42041935 were from Merck (Darmstadt, Germany). Antibodies for β-actin, VEGFR-2, HIF-1α (WB), hydroxy-HIF-1α (Pro564), phospho-eIF-4E (Ser209), eIF-4E, phospho-4E-BP1 (Ser65) and 4E-BP1 as well as horseradish peroxidase-conjugated anti-rat antibody were from Cell Signaling Technology (Danvers, MA, USA). Horseradish peroxidase-conjugated anti-rabbit antibody and the Aurum Total RNA Isolation Kit were from Bio-Rad Laboratories (Hercules, CA, USA). Antibodies for phospho-p70S6K (Thr389) and p70S6K were from Abcam (Cambridge, MA, USA). Antibodies for HIF-1α (IP), pVHL, S1P_1–3_, RACK1 and Hsp90, normal mouse IgG, Protein A/G PLUS-agarose beads and MG-132 were from Santa Cruz Biotechnology (Santa Cruz, CA, USA). CAY10444, and S1P_1_ and S1P_3_ antibodies were also purchased from Cayman Chemicals (Ann Arbor, MI, USA). Small interfering RNA (siRNA) for S1P_1–3_, PKCα and PKCβI and control siRNA were from Dharmacon Inc. (Lafayette, CO, USA). Another control and S1P_2_ siRNA were purchased from Ambion (Austin, TX, USA). HIF-1α siRNA was from MWG (Ebersberg, Germany), HIF-1α, VEGF-A, AMF, TGFα, S1P_1_, S1P_2_, S1P_3_, PKCα, PKCβI and HPRT1 primers from TAG Copenhagen (Copenhagen, Denmark) and Universal Probe Library probes from Roche (Basel, Switzerland). The BCA Protein Assay Reagent kit was from Thermo Fisher Scientific (Rockford, IL, USA). Oligo(dT)_15_ primers, RNAsin inhibitor, CellTiter 96 AQueous One Solution and DualGlo were from Promega (Madison, WI, USA). GAPDH primers and probe were from Oligomer (Helsinki, Finland), Absolute QPCR Rox Mix from Abgene (Epsom, UK) and dNTPs from Finnzymes (Espoo, Finland). Nitrocellulose transfer membrane was from Whatman (Maidstone, UK). Optisafe Hisafe 3 scintillation cocktail [^3^H]thymidine (1 mCi/ml) and the Western Lightning Plus-ECL kit were from Perkin Elmer (Waltham, MA, USA). The Kapa Probe Fast qPCR kit was from Kapa Biosystems (Boston, MA, USA) and the HiPerFect and The Amaxa electroporation device and Amaxa Cell Line Optimazation Nucleofector Kit were from Lonza (Basel, Switzerland).

### Cell Culture

ML-1 human follicular thyroid cancer cells were a kind gift from Dr. Johann Schönberger (University of Rosenburg, Germany). They were cultured in DMEM supplemented with 10% Bovine Serum Albumin (FBS), 2 mM L-glutamine and 100 U/ml penicillin/streptomycin. FTC-133 human follicular thyroid cancer cells were from Banca Biologica e Cell Factory, National Institute for Cancer Research (Genova, Italy). They were grown in Ham’s medium and DMEM (1∶1) supplemented with 10% FBS, 2 mM L-glutamine and 100 U/ml penicillin/streptomycin. Cells were cultured at 37°C in a water-saturated atmosphere containing 5% CO_2_ and 95% air. During hypoxia experiments cells were incubated in an In vivo2 hypoxia workstation (Ruskinn Technology, Bridgend, UK) with 1% oxygen at 37°C. Before treatment with S1P, cells were lipid-starved in medium containing 5% charcoal/dextran treated FBS (lipid-stripped FBS). For migration experiments cells were serum-starved in medium containing 0.2% fatty acid-free BSA (serum-free medium).

### Western Blotting

Cells were lipid-starved overnight before treatment. Whole cell lysates were obtained and Western blotting performed according to a protocol described elsewhere [17]. Proteins were detected with enhanced chemiluminescence using the Western Lightning Plus-ECL kit. Hsc70 or β-actin was used as a loading control. Levels of phosphorylated or hydroxylated proteins were normalized with the non-phosphorylated or non-hydroxylated form and with the loading control. Densitometric analysis of protein bands was done with the ImageJ program (http://rsbweb.nih.gov/ij/).

### Cell Migration and Haptotaxis

Cellular migration and haptotaxis was studied with 6.5 mm-diameter Transwell Permeable Support inserts with 8-µm pore size. The protocols have been described elsewhere [16], [17], [18].

### Transfection with siRNA

Transfection of siRNA was done with the N-TER or HiPerfect transfection reagents or by electroporation with a Gene Pulser Xcell electroporation system (Bio-Rad) (240 V, 975 µF) or with an Amaxa electroporation device and Amaxa Cell Line Optimization Nucleofector Kit according to the manufacturer’s instructions. 100 nM siRNA was used with N-TER, 5–20 nM with HiPerfect and 2 µM with electroporation. Down-regulation of target protein was approximately 50% for S1P_1_, 30% for S1P_2_, 60% for S1P_3_, 30% for PKCα, 50% for PKCβI and 35–90% for HIF-1α [approximately 35% in migration experiments done with N-TER and 90% in later experiments done with HiPerFect or electroporation (Fig. S5)]. Down-regulation of target mRNA (with HiPerfect or electroporation) was approximately 70% for S1P_1_, 65% for S1P_2_, 60% for S1P_3_, 35% for PKCα, 60% for PKCβI and 85% for HIF-1α (Fig. S6).

### Proliferation

Cellular proliferation was studied with a [ ^3^H]thymidine incorporation assay. Cells were lipid-starved overnight before treatment and the experiments were performed according to a protocol described elsewhere [16], [17].

### RNA Extraction, Reverse Transcriptase PCR and Quantitative Real-time PCR

RNA was isolated using the Aurum Total RNA Mini kit and RNA concentrations were determined using the RiboGreen RNA Quantitation Reagent. Reverse transcriptase PCR was performed with SuperScript III Reverse Transcriptase to produce cDNA. The quantitative PCR assays for HIF-1α, VEGF-A, AMF, TGFα and HPRT1 were designed using the Universal ProbeLibrary Assay Design Center (www.roche-applied-science.com). GAPDH and HPRT1 were used as reference genes. The primer and probe information are in Table S1. Reaction mixtures were prepared with ABsolute QPCR Rox Mix or with the KAPA Probe Fast qPCR Kit and real-time quantitative PCR was performed using the Applied Biosystems 7900HT Fast Sequence Detection System or the StepOnePlus Real-Time PCR system. The amplification results were analyzed with the SDS and RQ Manager programs (Applied Biosystems).

### Luciferase Assays

Cells were co-transfected with a total of 20 µg of either TK-Luc or HRE-Luc plasmid together with a Ubi-Renilla plasmid. The HRE-Luc plasmid was from Addgene (plasmid 26731; [49]. The TK-Luc and HRE-Luc plasmids contain a TK or HRE promoter and the firefly luciferase gene whereas Ubi-Renilla contains the Ubi promoter and the Renilla Reniformis luciferase gene. Firefly luciferase luminescence was normalized with Renilla luciferase luminescence. Transfection was done with an Amaxa electroporation device and Amaxa Cell Line Optimization Nucleofector Kit according to the manufacturer’s instruction. 24 h after transfection the cells were lipid-starved and the next day treated with S1P (100 nM) or CoCl_2_ (150 µM) for 7 h. Luminescence was measured with the DualGlo Luciferase Assay System according to the manufacturer’s instructions.

### Immunoprecipitation

Lysates for immunoprecipitation (IP) were made with IP lysis buffer (50 mM Tris-HCl pH 7.5, 150 mM NaCl, 0.1% NP-40, 0.2 mM PMSF, 0.5 µg/ml leupeptin). Lysates were adjusted to equal protein amount and volume and pre-cleaned with 20 µl of Protein A/G PLUS-agarose beads for 1 h at 4°C. Pre-cleaned lysates were incubated with 2 µg of antibody or IgG control overnight at 4°C and the next day incubated with 40 µl of Protein A/G PLUS-agarose beads for 2 h at 4°C. The agarose beads were washed five times with IP washing buffer (50 mM Tris-HCl pH 7.5, 250 mM NaCl, 0.1% NP-40), Laemmli sample buffer was added and the samples boiled.

### Statistical Analysis

HIF-1α half-lives were determined with a non-linear curve fit of Chx chase data using the one phase exponential decay equation. Half-lives were calculated as ln(2)/k, where k is the rate constant, and compared with an extra sum-of-squares F test. For other experiments the data is presented as mean ± SEM for at least three independent experiments and either Student’s t-test, one-way ANOVA with Dunnett’s *post hoc* test or one-way ANOVA with Bonferroni’s *post hoc* test was used for statistical analysis. Analysis was performed and graphs were created with the GraphPad Prism 4 program (San Diego, CA, USA).

## Results

### S1P is a Non-hypoxic Regulator of HIF-1α Expression

**Figure 1:**
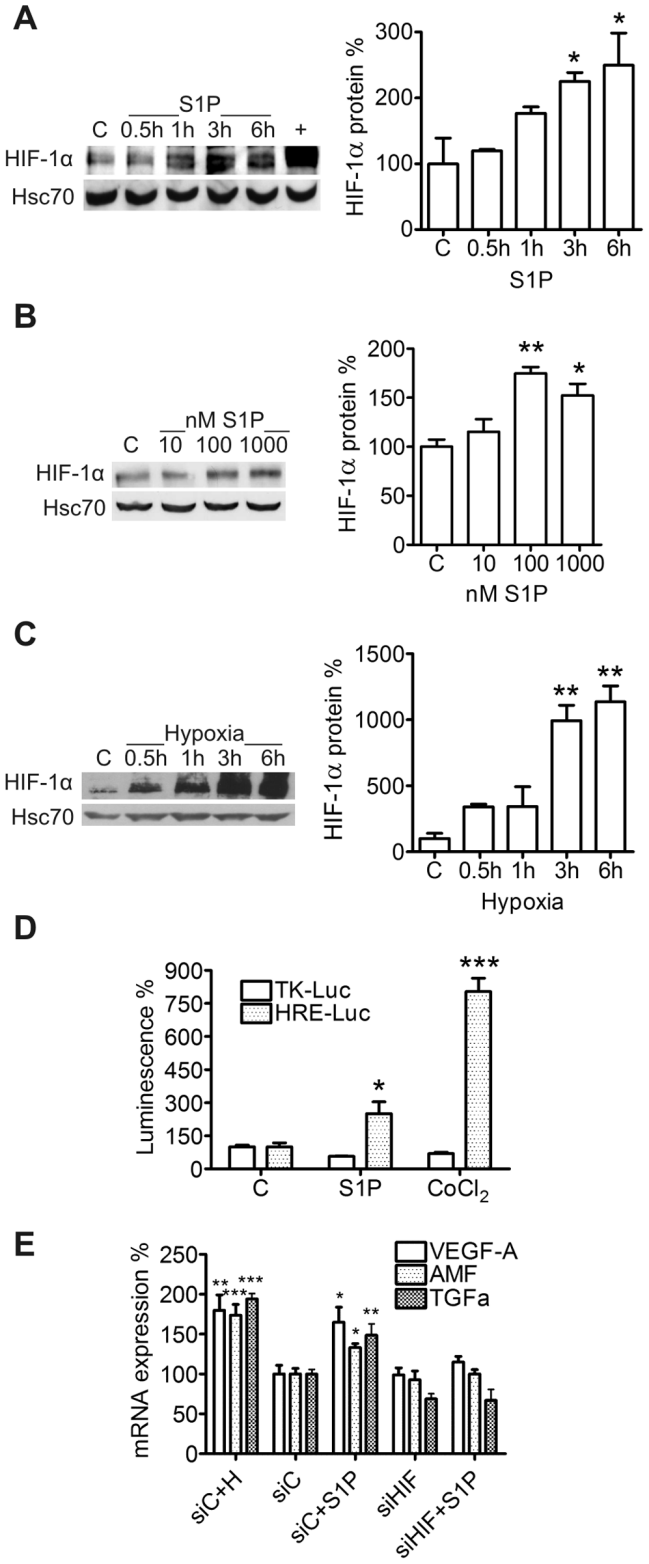
S1P increases HIF-1α protein expression and HIF-1 activity in ML-1 cells. (**A–B**) S1P increases HIF-1α expression in a time- and concentration dependent manner. Cells were stimulated with 100 nM S1P for the indicated times or with the indicated concentrations for 6 h. A lysate of CoCl_2_-treated cells was used as positive control for HIF-1α (+). (**C**) Hypoxia increases HIF-1α protein expression. Cells were incubated in hypoxia (1% O_2_) for the indicated times. (**D**) S1P increases expression from promoters containing the HRE sequence. ML-1 cells were transfected with a HRE-Luc or a negative control TK-Luc plasmid and treated with S1P (100 nM) or CoCl_2_ (150 µM) for 7 h. (**E**) S1P increases expression of HIF-1 target genes in a HIF-1α-dependent manner. ML-1 cells were transfected with control siRNA (siC) or siRNA against HIF-1α (siHIF) and treated with S1P (100 nM) or incubated in hypoxia (H, 1% O_2_) for 9 h. Hypoxia-treated samples were used as a positive control for HIF-1 target gene expression. Results are mean ± SEM, n ≥ 3. *P < 0.05, **P < 0.01 and ***P < 0.001 indicate statistically significant difference between S1P, COCl_2_ or hypoxia treatment and respective vehicle or siRNA control.

Since S1P treatment of ML-1 thyroid cancer cells strongly increases their migration [16] and HIF-1 is a known regulator of invasion and metastasis [7], [8], we investigated whether S1P could affect expression of the regulatory HIF-1α subunit in ML-1 cells. We found that S1P up-regulated HIF-1α protein in a time- and concentration dependent manner in normoxic conditions (Figs. 1A and 1B). As expected, hypoxia (1% O_2_) up-regulated HIF-1α in ML-1 cells (Fig. 1C). Hypoxia-induced HIF-1α expression was stronger than S1P-induced expression but the kinetics of HIF-1α increase was similar in both cases. S1P did not affect HIF-2α protein expression (results not shown). To determine whether S1P-induced HIF-1α expression is a common feature in follicular thyroid cancer cells, we treated FTC-133 cells with S1P. S1P up-regulated HIF-1α in a time-dependent manner in these cells also (Fig. S1A).

### S1P Increases HIF-1 Activity

Promoters of HIF-1 target genes contain a hypoxia response element (HRE) sequence to which HIF-1 binds [7], [8]. To investigate whether S1P increases expression of such genes, we performed luciferase assays with cells transfected with a HRE-Luc plasmid construct. S1P and CoCl_2_, which was used as a positive control, significantly increased luciferase activity of HRE-Luc cells (Fig. 1D). The effect was HRE-specific since neither S1P nor CoCl_2_ could increase luciferase activity of cells transected with a TK-Luc plasmid. We also determined whether S1P treatment induced expression of known HIF-1 target genes and used hypoxia as a positive control. S1P significantly increased mRNA expression of VEGF-A, autocrine motility factor (AMF) and transforming growth factor-α (TGFα) (Fig. 1E). Importantly, knockdown of HIF-1α with siRNA abolished the S1P-induced expression of these genes.

### S1P Induces HIF-1α Expression via S1P_3_, G_i_, PKCβI, MEK, PI3K and mTOR

**Figure 2:**
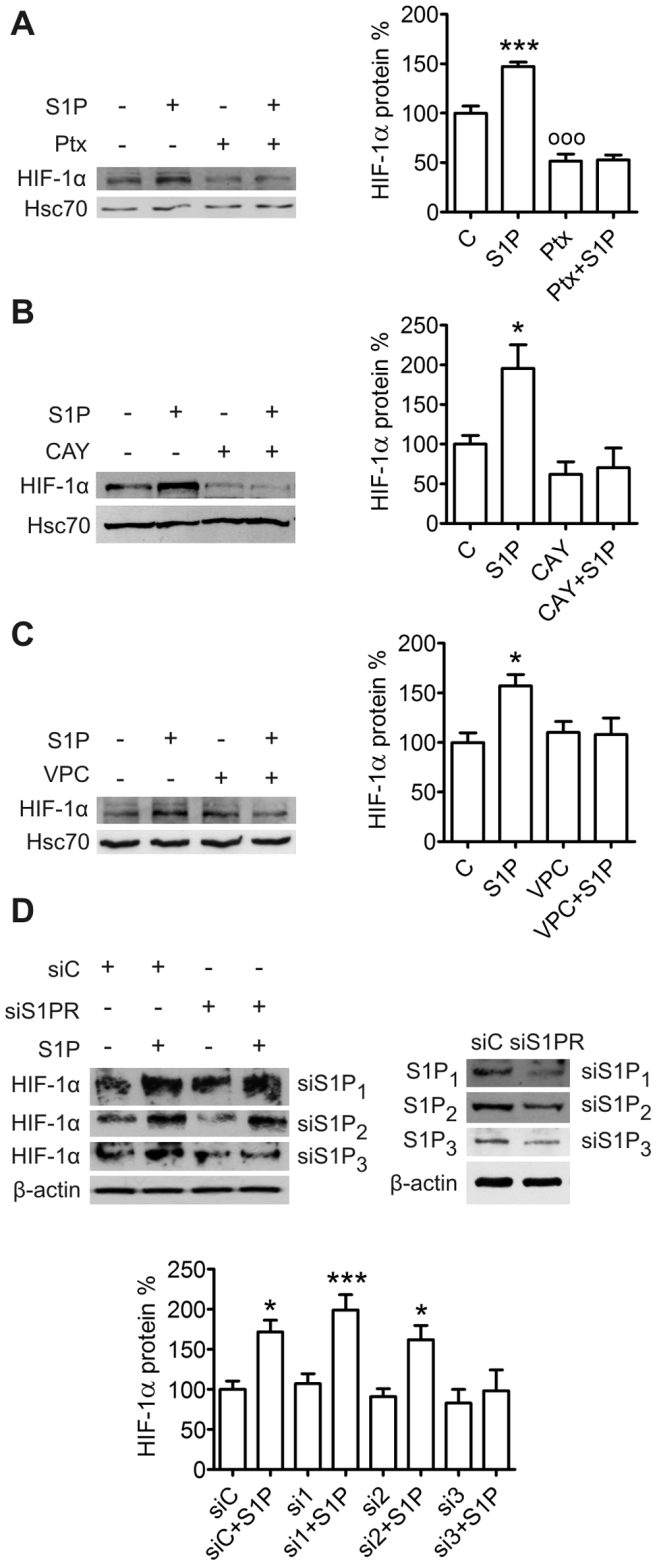
S1P up-regulates HIF-1α via S1P_3_ and G_i_ in ML-1 cells. Inhibition of (**A**) G_i_ proteins, (**B**) S1P_3_ or (**C**) S1P_1_ and S1P_3_ and (**D**) knockdown of S1P_3_ prevents S1P-induced HIF-1α expression. Cells were pretreated with Pertussis toxin (Ptx, 100 ng/ml, 24 h), CAY10444 (CAY, 10 µM, 1 h) or VPC-23019 (VPC, 10 µM, 30 min) or transfected with control siRNA (siC) or S1P receptor siRNA (si1-3) and stimulated with S1P (100 nM) for 6 h. Results are mean ± SEM, n ≥ 3. *P < 0.05 and ***P < 0.001 indicate statistically significant difference between S1P treatment and respective vehicle or siRNA control, ^ooo^P < 0.001 indicates statistically significant difference between inhibitor treatment and vehicle control.

ML-1 cells express S1P receptors 1,2,3 and 5 (S1P_1–3,5_) and their migration is regulated via S1P_1,3_ and G_i_ proteins [16]. Pretreatment with the G_i_ inhibitor Pertussis toxin (Ptx, 100 ng/ml, 24 h) both decreased the basal level of HIF-1α and prevented S1P-induced expression (Fig. 2A). Pretreatment with the S1P_3_ inhibitor CAY10444 [19] (10 µM, 1 h) or with the S1P_1,3_ antagonist VPC-23019 (1 µM, 30 min) also abolished the S1P-evoked increase (Figs. 2B and 2C) while S1P_1_ agonist SEW-2871 (1µM) was without an effect on HIF-1α expression (Fig. S2). Accordingly, down-regulation of S1P_1_ and S1P_2_ (by approximately 50% and 30% respectively) with siRNA did not attenuate S1P-induced expression of HIF-1α whereas down-regulation of S1P_3_ (by approximately 60%) abolished the effect (Fig. 2D).

**Figure 3:**
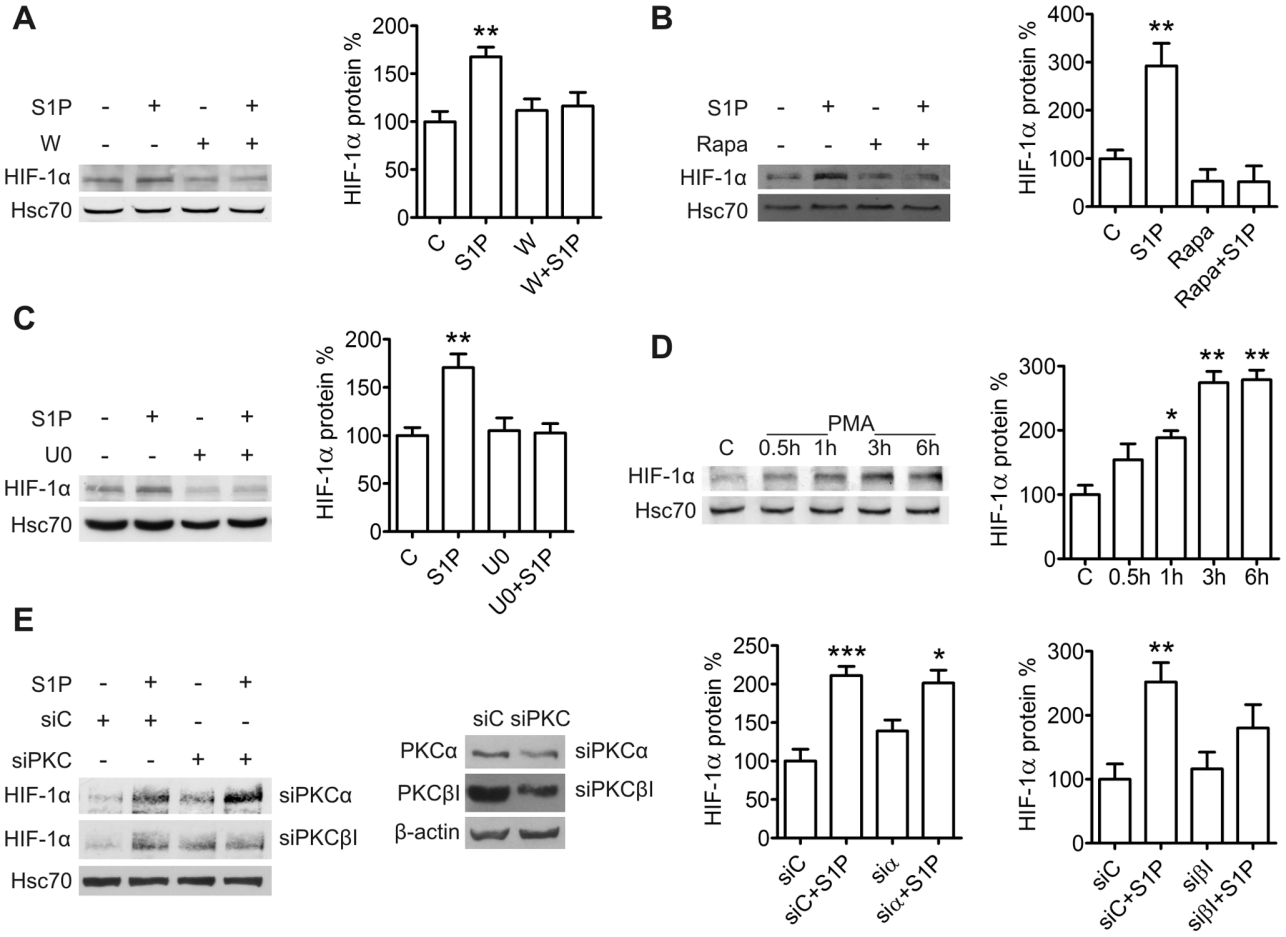
S1P up-regulates HIF-1α via PI3K, mTOR, MEK and PKCβI in ML-1 cells. Inhibition of (**A**) PI3K, (**B**) mTOR and (**C**) MEK prevents S1P-induced HIF-1α expression. Cells were preincubated with wortmannin (W, 10 µM, 30 min), rapamycin (Rapa, 100 ng/ml, 1h) or with U0126 (U0, 10 µM, 1 h) and stimulated with S1P (100 nM) for 6 h. (**D**) Phorbol 12-myristate 13-acetate (PMA) induces HIF-1α expression. Cells were stimulated with PMA (100 nM) for the indicated times. (**E**) PKCβI mediates S1P-induced HIF-1α expression transfected with PKC isoform specific siRNA and stimulated with S1P (100 nM) for 6 h. Results are mean ± SEM, n ≥ 3. *P < 0.05, **P < 0.01 and ***P < 0.001 indicate statistically significant difference between S1P treatment and respective vehicle or siRNA control.

Since both the MEK/ERK and PI3K/Akt/mTOR pathway lie downstream of S1P receptors [3], [4] and are involved in regulation of HIF-1α expression [7], we investigated their involvement in S1P-induced up-regulation of HIF-1α. Pretreatment of ML-1 cells with the PI3K inhibitor wortmannin (10 µM, 30 min) or the mTOR inhibitor rapamycin (100 ng/ml, 1 h) prevented the effect of S1P (Figs. 3A and 3B), as did pretreatment with the MEK inhibitor U0126 (10 µM, 1 h) (Fig. 3C).

S1P treatment induces translocation of PKCα and βI to the membrane fraction in ML-1 cells [20]. We investigated whether these isoforms also mediate S1P-induced HIF-1α expression. Treatment with the potent PKC activator phorbol 12-myristate 13-acetate (PMA, 100 nM) induced HIF-1α protein expression (Fig. 3D). Down-regulation of PKCα (by approximately 30%) with siRNA was not able to prevent S1P-induced expression. In contrast, down-regulation of PKCβI (by approximately 50%) did not affect basal expression but abolished the effect of S1P on HIF-1α (Fig. 3E).

These results show that S1P stimulates HIF-1α expression via S1P_3_ and G_i_ and their downstream effectors PKCβI, MEK, PI3K and mTOR and in ML-1 cells.

### Effect of S1P on HIF-1α Synthesis and Stability

**Figure 4:**
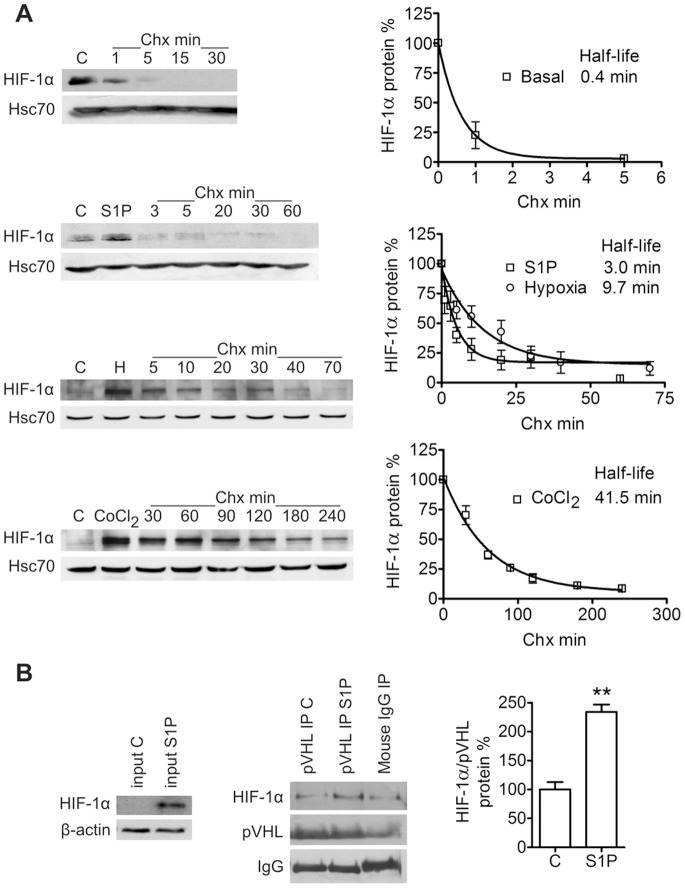
S1P stabilizes HIF-1α independently of pVHL binding. (**A**) S1P prolongs HIF-1α half-life. Cells were either left untreated, treated with S1P (100 nM) for 6 h, incubated in hypoxia (1% O_2_) for 6 h or treated with CoCl_2_ (150 µM) for 3 h before the cycloheximide chase (Chx, 5 µg/ml). S1P, hypoxic conditions or CoCl_2_ were present throughout the chase. Time points are mean ± SEM, n = 3–10. Curve fit was done with the one phase exponential decay equation. (**B**) S1P does not inhibit binding of pVHL to HIF-1α. Cells were treated with S1P (100 nM) for 6 h. The level of co-immunoprecipitated HIF-1α was compared with the level of immunoprecipitated pVHL and IgG bands were used as a loading control. **P < 0.01 indicates statistically significant difference between S1P treatment and vehicle control.

We attempted to determine whether S1P increases synthesis or stability of HIF-1α. The HIF-1α protein is up-regulated by S1P within 3 h but we saw no effect on HIF-1α mRNA during a 6-h treatment (Fig. S3A). Interestingly, we did see a small but significant increase in HIF-1a mRNA after 9 h of S1P treatment (Fig. S3B). We performed a classical chase experiment with the translation inhibitor cycloheximide (Chx) in order to compare the half-lives of basal, S1P-, hypoxia-, and CoCl_2_-induced HIF-1α (Fig. 4A). Half-life of basal HIF-1α was significantly lower than that of S1P-induced HIF-1α (0.4 and 3.0 min, respectively, **P < 0.01 with an extra sum-of-square’s F test), indicating that S1P increases HIF-1α stability. Hypoxia and CoCl_2_ were used as controls which are known to stabilize HIF-1α. Accordingly, half-lives of hypoxia- and CoCl_2_-induced HIF-1α were high (9.7 and 41.5 min, respectively). We attempted to use [ ^35^S]methionine pulse-chase labeling as an additional method to determine HIF-1α half-lives but were not able to immunoprecipitate sufficient amounts of labeled HIF-1α. When cells were pretreated with proteasome inhibitor MG-132 (20 µM, 1 h) to prevent HIF-1α degradation, S1P was not able to elevate the HIF-1α level (Fig. S4A) also suggesting that S1P may affect HIF-1α stability. However, the approximately two-fold increase caused by S1P may have been lost during the over tenfold up-regulation seen in response to MG-132 treatment.

Instability of the HIF-1α protein in normoxia is primarily due to its oxygen-dependent hydroxylation on prolines 402 and 564 [9]–[11]. We used hydroxy-HIF-1α-specific antibodies to study HIF-1α hydroxylation status. S1P treatment decreased the fraction of Pro402-hydroxylated HIF-1α whereas the fraction of Pro564-hydroxylated HIF-1α remained unchanged (Figs. S4B and S4C). We saw the same S1P-induced decrease of Pro402-hydroxylated HIF-1α in comparison to total HIF-1α in FTC-133 cells (Fig S1E). However, co-immunoprecipitation of HIF-1α with pVHL in ML-1 cells showed that even S1P-evoked HIF-1α was bound by pVHL (Fig. 4B). The basal stability of HIF-1α is controlled by binding of RACK1 and Hsp90. Pretreatment with Hsp90 inhibitor 17-AAG (2 µM, 16 h) abolished the S1P-induced HIF-1α expression (Fig. S4D). To verify the result we attempted to co-immunoprecipitate HIF-1α from S1P-treated lysates with RACK1 and Hsp90 antibodies but could not detect any co-immunoprecipitated HIF-1α (Fig. S4E).

**Figure 5:**
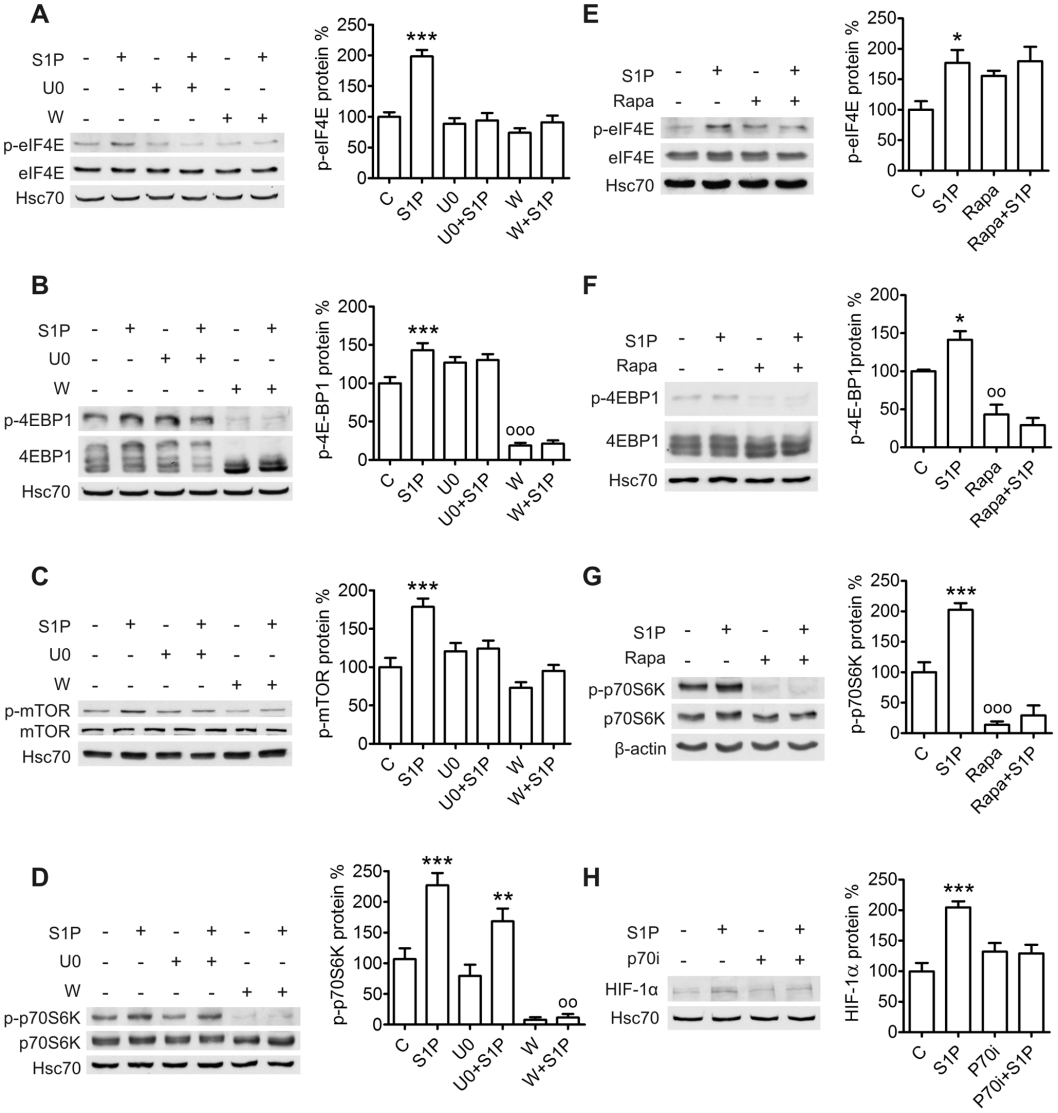
S1P activates translational regulators in ML-1 cells. (**A–D**) S1P induces phosphorylation of mTOR, eIF-4E and 4E-BP1 via PI3K and MEK and phosphorylation of p70S6K via PI3K. Cells were preincubated with wortmannin (W, 10 µM, 30 min) or U0126 (U0, 10 µM, 1 h) and stimulated with S1P (100 nM) for 30 min. (**E–G**) S1P induces phosphorylation of eIF-4E, 4E-BP1 and p70S6K via mTOR. Cells were preincubated with rapamycin (Rapa, 100 ng/ml, 1 h) and stimulated with S1P (100 nM) for 30 min. (**H**) Inhibition of p70S6K prevents S1P-induced HIF-1α expression. Cells were preincubated with p70S6K inhibitor (p70i, 10 µM, 1 h) and stimulated with S1P (100 nM) for 6 h. Results are mean ± SEM, n ≥ 3. *P < 0.05, **P < 0.01 and ***P < 0.001 indicate statistically significant difference between S1P treatment and respective vehicle control, ^oo^P < 0.01 and ^ooo^P < 0.001 indicate statistically significant difference between inhibitor treatment and vehicle control.

We also studied the effect of S1P on translational regulators known to be involved in HIF-1α protein synthesis: eIF-4E and p70S6K [7], [15]. eIF-4E function is regulated by stimulatory phosphorylation and binding of the inhibitor 4E-BP1 [21], [22]. Phosphorylation of 4E-BP1 on multiple residues dissociates it from eIF-4E [21], [23]. p70S6K activity also requires phosphorylation of several residues [25]–[28]. We found S1P treatment of ML-1 cells to induce a rapid phosphorylation of eIF-4E on Ser209, 4E-BP1 on Ser65 and p70S6K on Thr389, all residues implicated in eIF-4E or p70S6K activation. Accordingly, S1P also induced rapid phosphorylation of mTOR which lies upstream of these proteins. Pretreatment with U0126 or wortmannin prevented mTOR phosphorylation (Fig. 5C), pretreatment with U0126, wortmannin or rapamycin prevented eIF-4E and 4E-BP1 phosphorylation (Figs. 5A, 5B, 5E and 5F) and pretreatment with wortmannin or rapamycin prevented p70S6K phosphorylation (Figs. 5D and 5G). Wortmannin prevented hyperphosphorylation of 4E-BP1 altogether which is consistent with the sequential nature of the phosphorylations [24]. While U0126 did not affect S1P-induced p70S6K phosphorylation, both wortmannin and rapamycin strongly reduced basal phospho-p70S6K levels indicating the importance of the PI3K/Akt/mTOR pathway as a regulator of p70S6K in ML-1 cells. Furthermore, preincubation with an inhibitor of p70S6K (10 µM, 1 h) prevented S1P-induced HIF-1α expression (Fig. 5H). S1P induced p70S6K and eIF4E phosphorylation also in FTC-133 cells (Fig. S1C–D). However, when we transfected ML-1 cells with in vitro-transcribed mRNA containing the 5′ untranslated region (5′-UTR) of murine HIF-1α followed by the firefly luciferase gene, we could not detect a S1P-induced increase in luciferase activity (result not shown).

Taken together these results provide evidence for a S1P-induced effect on both synthesis and stability of HIF-1α. It is possible that both mechanisms are involved. Also, although HIF-1α transcription is not responsible for the initial HIF-1α up-regulation, it may mediate prolonged HIF-1α expression.

### HIF-1α is Involved in Basal and S1P-induced ML-1 Migration

**Figure 6:**
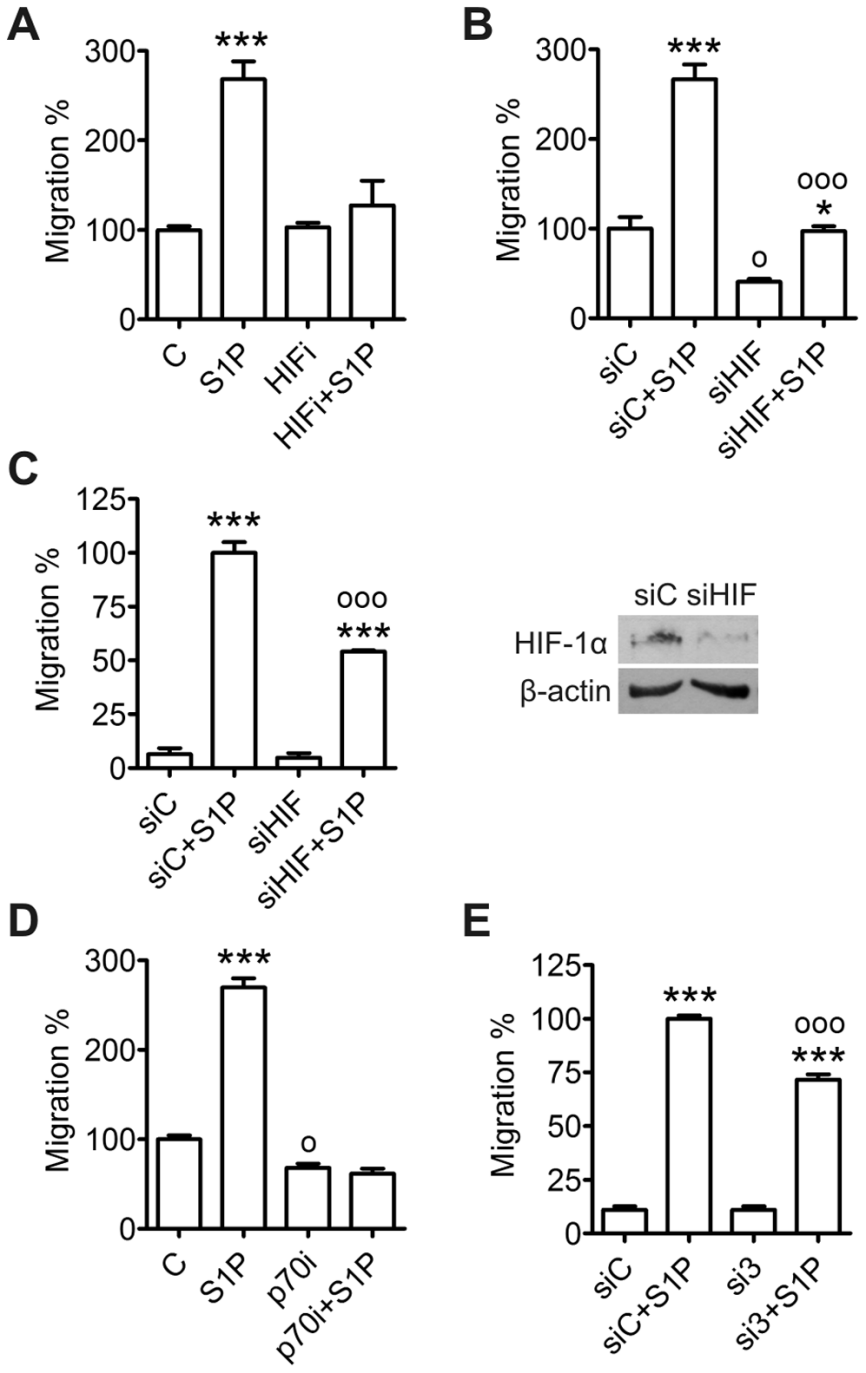
HIF-1α mediates basal and S1P-induced migration of ML-1 cells. (**A**) Inhibition of HIF-1 attenuates S1P-induced migration. Cells were preincubated with HIF-1 inhibitor (HIFi, 10 µM, 30 min) and S1P (100 nM, 30 min) and allowed to migrate towards serum for 8 h. (**B**) Down-regulation of HIF-1α decreases basal migration. Cells were transfected with HIF-1α siRNA and allowed to migrate towards serum and S1P (100 nM) for 8 h. (**C**) Down-regulation of HIF-1α attenuates S1P-induced migration. Cells were transfected with HIF-1α siRNA and allowed to migrate towards S1P (100 nM) for 20 h. (**D**) Inhibition of p70S6K decreases basal migration and prevents S1P-induced migration. Cells were preincubated with p70S6K inhibitor (p70i, 10 µM, 30 min) and S1P (100 nM, 30 min) and allowed to migrate towards serum for 8 h. (**E**) Down-regulation of S1P_3_ attenuates S1P-induced migration. Cells were transfected with S1P_3_ siRNA and allowed to migrate towards serum and S1P (100 nM) for 8 h. Results are mean ± SEM, n ≥ 3. *P < 0.05 and ***P < 0.001 indicate statistically significant difference between S1P treatment and respective vehicle or siRNA control, ^o^P < 0.05 and ^ooo^P < 0.001 indicate statistically significant difference between siRNA treatment and control siRNA, between siRNA+S1P treatment and control siRNA+S1P or between inhibitor treatment and vehicle control.

Since S1P treatment increased HIF-1α expression in ML-1 cells, we determined whether this up-regulation is involved in regulating the S1P-induced migration. Preincubation with a HIF-1 inhibitor [29] (10 µM, 30 min) strongly attenuated S1P-induced migration (Fig. 6A). HIF-1α siRNA lowered basal migration when serum was used as a chemoattractant (Fig. 6B) and S1P-induced migration when S1P alone was used as a chemoattractant (Fig. 6C). HIF-1α siRNA also decreased migration of FTC-133 cells towards S1P (Fig. S1B). Interestingly, preincubation with a p70S6K inhibitor completely abolished S1P-induced migration (Fig. 6D). S1P_3_ siRNA attenuated S1P-induced ML-1 migration (Fig. 6E) as did S1P_3_ inhibitor CAY10444 (result not shown).

**Figure 7:**
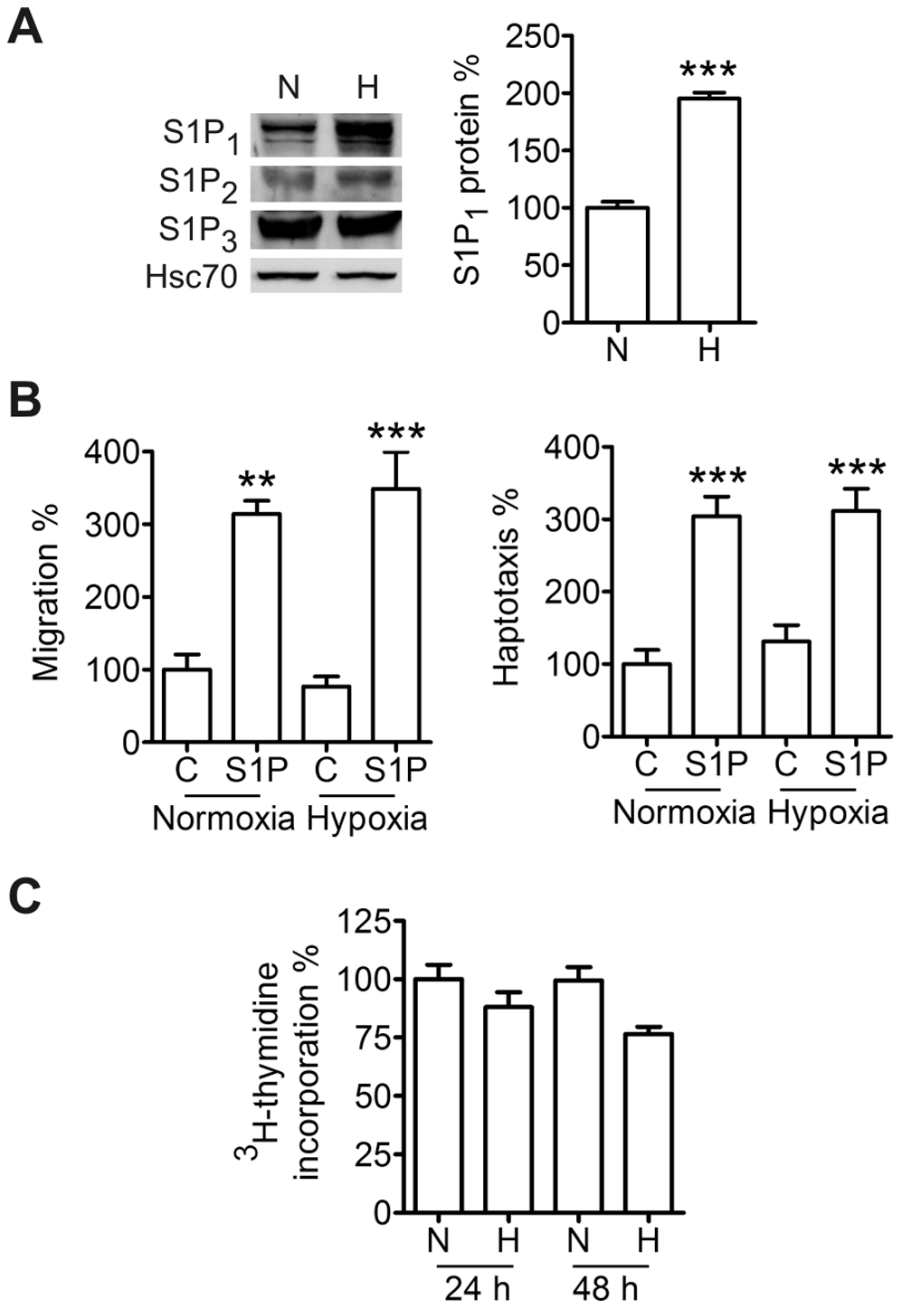
Hypoxia up-regulates S1P_1_ but does not affect ML-1 migration. (**A**) Hypoxia increases S1P_1_ protein expression. Cells were incubated in hypoxia (1% O_2_) for 24 h. (**B**) Hypoxia does not affect basal or S1P-induced migration or haptotaxis. Cells were allowed to migrate in normoxia or hypoxia (1% O_2_) towards serum and S1P in the migration experiments or towards collagen and S1P in the haptotaxis experiments for 8 h. (**C**) Hypoxia does not affect proliferation. Cells were incubated in normoxia or hypoxia (1% O_2_) for the indicated times. Results are mean ± SEM, n ≥ 3. **P < 0.01 and ***P < 0.001 indicate statistically significant difference between S1P treatment and respective control.

We also conducted migration experiments in hypoxia. We determined whether hypoxia could affect expression of the S1P receptors controlling migration. S1P_1_ protein expression was increased in hypoxic conditions while S1P_2_ and S1P_3_ were not affected (Fig. 7A). However, hypoxia did not increase basal or S1P-induced migration or haptotaxis (Fig. 7B). Changes in proliferation did not interfere with the migration experiments since hypoxia did not decrease ML-1 proliferation during a 48-h incubation (Fig. 7C).

Thus, we conclude that HIF-1α regulates basal ML-1 migration and, in part, S1P-induced migration. However, hypoxia *per se* does not affect ML-1 migration.

## Discussion

In the current study we identify S1P as a non-hypoxic inducer of HIF-1α expression in thyroid cancer cells. We show that S1P increases HIF-1 activity and that HIF-1 mediates S1P-induced cell migration. We also present putative signaling pathways leading from extracellular S1P to increased HIF-1α.

Several studies have shown hypoxia to increase sphingosine kinase expression and activity [12], [30]–[33] and according to Ader *et al.* [12], SK1 regulates hypoxia-induced stabilization of HIF-1α via Akt and GSK3. S1P has also been shown to regulate HIF-1α transcription in mouse T cells [34] and macrophages [35]. The most relevant studies in comparison to our work are the identification of S1P as a non-hypoxic regulator of HIF-1α expression in vascular endothelial and smooth muscle cells [36] and in HepG2 liver carcinoma cells [37]. The focus of the former study was on the regulatory role of S1P and HIF-1α in the vascular system. In vascular cells S1P increased stability of the HIF-1α protein via activation of the anti-migratory S1P_2_ but independently of G_i_ proteins. In contrast, S1P-induced HIF-1α expression in ML-1 thyroid cancer cells is mediated by the pro-migratory S1P_3_ as well as G_i_. In the latter study the focus was on identification of a S1P derivative (NHOBTD) and its effect on angiogenesis. They show S1P to increase HIF-1α expression in HepG2 cells and NHOBTD to prevent both S1P-induced HIF-1α up-regulation and S1P-induced VEGF secretion presumably mediated by HIF-1. In comparison to these studies we have also investigated signaling pathways mediating HIF-1α up-regulation and show S1P-induced HIF-1α expression to have a functional outcome in increased migration.

Burrows *et al.* [38] have compared basal and hypoxia-induced HIF-1α expression levels in normal thyroid tissues, primary thyroid tumors and thyroid cancer cell lines, including the follicular WRO and FTC-133 cell lines. They showed HIF-1α expression to be elevated in thyroid carcinomas and to correlate with malignancy, making it a potential target for thyroid cancer therapy. That we now identify S1P as a non-hypoxic regulator of HIF-1α in follicular ML-1 and FTC-133 cells suggests that S1P-induced HIF-1α expression may be involved in thyroid tumor formation and cancer progression.

One central aim of the current study was to investigate the signaling leading to HIF-1α regulation. We found S1P_3_ and G_i_ to mediate S1P-induced HIF-1α expression via PKCβI, MEK, PI3K and mTOR. Additionally, we show S1P to activate translational regulators eIF-4E and p70S6K. While the MEK/ERK and PI3K/Akt/mTOR cascades are known to regulate HIF-1α translation [7], PKC has been implicated in controlling HIF-1α transcription [39]. However, the initial S1P-induced HIF-1α up-regulation in ML-1 cells was not due to increased transcription. We have previously shown S1P-induced ERK1/2 phosphorylation in ML-1 cells to be mediated by PKCα rather than PKCβI [20]. Therefore, the exact role of PKCβI in S1P-induced HIF-1α expression remains unknown. Zhang *et al.* [40] showed nicotine-induced HIF-1α accumulation to be mediated by classical PKC isoforms as well as phosphorylation of Akt, ERK, 4E-BP1 and p70S6K in lung cancer cells. Therefore, nicotine may regulate HIF-1α expression in a similar PKC-dependent manner in lung cancer cells as S1P does in ML-1 cells. The signaling behind S1P-evoked HIF-1α also resembles IGF-1-induced HIF-1α expression in colon cancer cells and angiotensin II-evoked HIF-1α expression in vascular smooth muscle cells [39], [41]. Whether phosphorylation of eIF-4E actually activates it has been a controversial subject [21], [22], [42]–[44] but nonetheless, phosphorylation of 4E-BP1 is sufficient to activate eIF-4E [21].

We performed numerous experiments in order to determine whether S1P regulates HIF-1α synthesis or stability. According to protein half-life measurements S1P treatment stabilizes HIF-1α. The half-life of basal normoxic HIF-1α is commonly considered to be approximately 5 min but in ML-1 cells this half-life was as low as 0.4 min. Moroz *et al.* [45] have studied kinetics of HIF-1α degradation and showed the half-life of normoxic HIF-1α to be 3–6 min in their cell lines. Obviously, exact protein half-life is cell line specific. Half-lives of S1P-, hypoxia- and CoCl_2_-induced HIF-1α reflect the level of HIF-1α up-regulation seen in ML-1 cells: hypoxia and CoCl_2_ are several fold stronger inducers of HIF-1α expression in ML-1 cells than S1P. We saw a S1P-induced decrease in Pro402-hydroxylation, which did not however inhibit binding of pVHL to HIF-1α. This is not necessarily contradictory since it has been shown that hydroxylation of either Pro402 or Pro564 is sufficient to promote pVHL binding [11]. Since Hsp90 inhibition prevented S1P-induced HIF-1α expression, HIF-1α stabilization might be mediated by decreased RACK1 binding and increased Hsp90 binding to HIF-1α. And as PI3K mediated S1P-evoked HIF-1α expression, the involvement of the Akt/GSK3 pathway is also possible. The signaling evoked by S1P in ML-1 cells is practically identical to the signaling induced by growth factors to increase HIF-1α translation through activation of p70S6K and eIF-4E. On the other hand, that S1P did not increase translation of mRNA containing the murine 5′-UTR of HIF-1α points to S1P not affecting HIF-1α synthesis. However, changes in HIF-1α translation will readily affect HIF-1α levels because of the protein’s low basal expression and short half-life whereas the effect on luciferase levels may not be as strong. Taken together, our data points to S1P stabilizing the HIF-1α protein but potentially also increasing its translation.

An important part of the project was to determine the significance of S1P-induced HIF-1α expression for the S1P-induced migration of ML-1 cells. We were able to attenuate basal and S1P-induced ML-1 migration by HIF-1α inhibition. As a control we also conducted experiments in hypoxia. Surprisingly, we did not see a significant increase in either migration or haptotaxis in hypoxia. Thus, other factors induced or inhibited by hypoxic stress may have counteracted the migratory effect. Interestingly, although hypoxia significantly elevated expression of the pro-migratory S1P_1_ receptor, S1P-induced migration was not increased either. However, hypoxia-induced up-regulation of S1P_1_ is consistent with this receptor being essential for S1P function in vascular development [46]–[48], and S1P_1_ expression being regulated via VEGF signaling in ML-1 cells [14].

**Figure 8:**
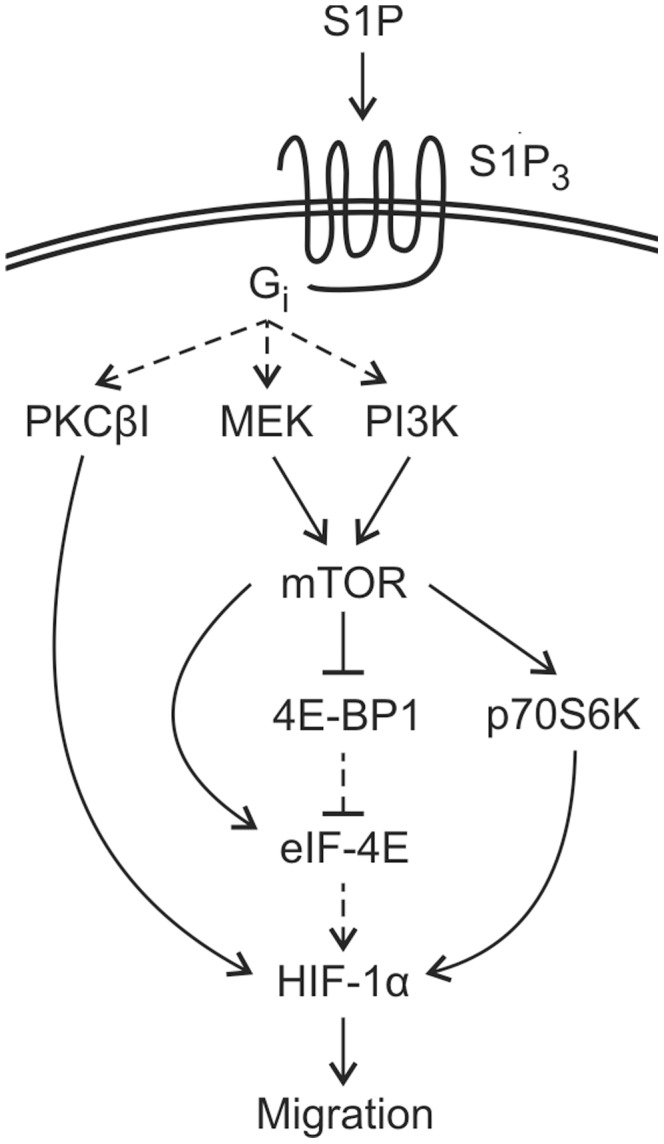
Schematic representation of the putative signaling involved in S1P-induced HIF-1α expression in ML-1 cells. S1P stimulation up-regulates the HIF-1α protein in normoxia. This effect is dependent on activity of S1P_3_ and G_i_ as well as their downstream effectors PKCβI, PI3K and MEK (S1P receptor signaling reviewed in 3 and 4). We suggest S1P to regulate both stability and translation of HIF-1α. S1P stimulation increases phosphorylation of mTOR via MEK and PI3K and phoshorylation of p70S6K, eIF-4E and 4E-BP1 via MEK and/or PI3K/mTOR and inhibition of p70S6K prevents S1P-induced up-regulation of HIF-1α. HIF-1 is involved in both basal and S1P-induced ML-1 migration.

In conclusion we identify S1P, a bioactive lipid readily available in blood, as a non-hypoxic regulator of HIF-1α expression in thyroid cancer cells. We show S1P to increase HIF-1 activity and to be a co-factor in S1P-induced migration. We also present signaling pathways involved in S1P-induced HIF-1α expression (Fig. 8). Altogether our work increases the knowledge of both the oncogenic function of S1P and normoxic regulation of HIF-1.

## Supporting Information

Figure S1 **S1P has similar effects on FTC-133 follicular thyroid cancer cells as on ML-1 cells.** (**A**) S1P up-regulates HIF-1α in FTC-133 cells. Cells were treated with S1P (100 nM) for the indicated times. (**B**) HIF-1α siRNA attenuates migration of FTC-133 cells towards S1P. Cells were transfected with HIF-1α siRNA and allowed to migrate towards S1P (100 nM) for 20 h. (**C-D**) S1P induces rapid phosphorylation of p70S6K and eIF4E in FTC-133 cells. Cells were treated with S1P (100 nM) for the indicated times. (**E**) S1P decreases the ratio of HIF-1α hydroxylated on Pro402 and total HIF-1α in FTC-133 cells. Cells were treated with S1P (100 nM) for the indicated times. Results are mean ± SEM, n ≥ 3. *P < 0.05, **P < 0.01 and ***P < 0.001 indicate statistically significant difference between S1P treatment and respective vehicle or siRNA control, ^ooo^P < 0.001 indicates statistically significant difference between control siRNA+S1P and HIF-1α siRNA+S1P.
(TIF)

Figure S2 **S1P_1_ activation does not increase HIF-1α expression in ML-1 cells.** Cells were treated with 10 µM SEW-2871 (SEW) for 6 h. Result is mean ± SEM, n = 6.
(TIF)

Figure S3 **S1P up-regulates HIF-1α mRNA only after long S1P incubation in ML-1 cells.** (**A**) The initial S1P-induced HIF-1α expression is not mediated by increased transcription. Cells were treated with S1P (100 nM) for the indicated times. (**B**) siRNA against HIF-1α caused an approximately 90% knockdown of HIF-1α mRNA. Cells were transfected with control siRNA (siC) or HIF-1α siRNA (siHIF) and treated with S1P (100 nM) or incubated in hypoxia (1% O_2_) for 9 h. Results are mean ± SEM, n ≥ 3. **P < 0.01 and ***P < 0.001 indicate statistically significant difference between S1P treatment and vehicle control, ^ooo^P < 0.001 indicates significant difference between HIF-1α siRNA and control siRNA.
(TIF)

Figure S4 **S1P may affect HIF-1α stability.** (**A**) Inhibition of proteasomes strongly elevates the basal HIF-1α protein level and S1P is not able to increase it further. Cells were pre-incubated with MG-132 (MG, 20 µM, 1 h) and stimulated with S1P (100 nM) for 6 h. (**B-C**) S1P inhibits hydroxylation of HIF-1α on Pro402 but does not inhibit hydroxylation of Pro564. Cells were treated with S1P (100 nM) for 6 h. (**D**) Inhibition of Hsp90 decreases basal HIF-1α expression and prevents S1P-induced up-regulation of HIF-1α. Cells were pre-incubated with 17-(allylamino)-17-desmethoxygeldanamycin (17-AAG, 2 µM, 16 h) and stimulated with S1P (100 nM) for 6 h. (**D**) RACK1 and Hsp90 may not bind to HIF-1α in ML-1 cells. Cells were treated with S1P (100 nM) for 6 h. Lysates were immunoprecipitated with a RACK1 or Hsp90 antibody or an IgG control. A lysate of CoCl_2_-treated cells was used as positive control for HIF-1α. Results are mean ± SEM, n ≥ 3. **P < 0.01 indicates statistically significant difference between S1P treatment and vehicle control, ^o^P < 0.05 and ^ooo^P < 0.001 indicate statistically significant difference between inhibitor treatment and vehicle control.
(TIF)

Figure S5 **HIF-1α siRNA caused a knockdown of approximately 90% (in the qPCR experiments) and prevented S1P-induced HIF-1α expression.** Cells were transfected with control siRNA (siC) or HIF-1α siRNA (siHIF) and treated with S1P (100 nM) for 6 h.
(TIF)

Figure S6 **qPCR results showing expression of targeted mRNAs.** siRNAs against S1P_1_, S1P_2_, S1P_3_ and PCKβI caused a knockdown of 60–70% and siRNA against PKCα caused a knockdown of approximately 35%. Results are mean ± SEM, n ≥ 5. **P < 0.01 and ***P < 0.001 indicate statistically significant difference between control siRNA and targeting siRNA.
(TIF)

Table S1 Primer information.
(DOC)
