# Supplementary figures and images for: Sphingosine-1-Phosphate as a Regulator of Hypoxia-Induced Factor-1α in Thyroid Follicular Carcinoma Cells
Source: PLoS One. 2013 Jun 18;8(6):e66189. doi: 10.1371/journal.pone.0066189 (PMC3688870; doi:10.1371/journal.pone.0066189)

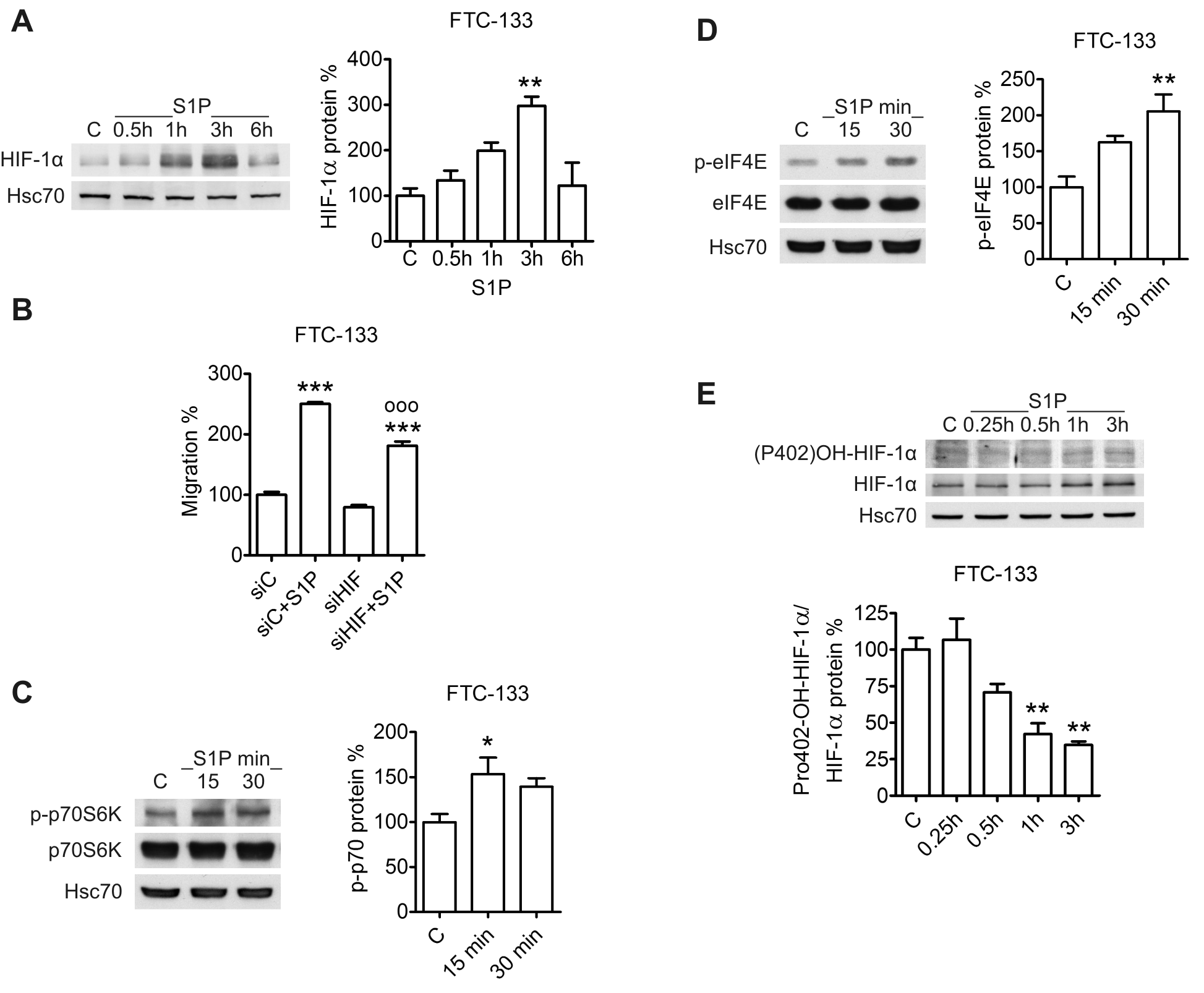

Supplement: Figure S1 — S1P has similar effects on FTC-133 follicular thyroid cancer cells as on ML-1 cells. (A) S1P up-regulates HIF-1α in FTC-133 cells. Cells were treated with S1P (100 nM) for the indicated times. (B) HIF-1α siRNA attenuates migration of FTC-133 cells towards S1P. Cells were transfected with HIF-1α siRNA and allowed to migrate towards S1P (100 nM) for 20 h. (C-D) S1P induces rapid phosphorylation of p70S6K and eIF4E in FTC-133 cells. Cells were treated with S1P (100 nM) for the indicated times. (E) S1P decreases the ratio of HIF-1α hydroxylated on Pro402 and total HIF-1α in FTC-133 cells. Cells were treated with S1P (100 nM) for the indicated times. Results are mean ± SEM, n ≥ 3. *P < 0.05, **P < 0.01 and ***P < 0.001 indicate statistically significant difference between S1P treatment and respective vehicle or siRNA control, oooP < 0.001 indicates statistically significant difference between control siRNA+S1P and HIF-1α siRNA+S1P. (TIF) [file pone.0066189.s001.tif]

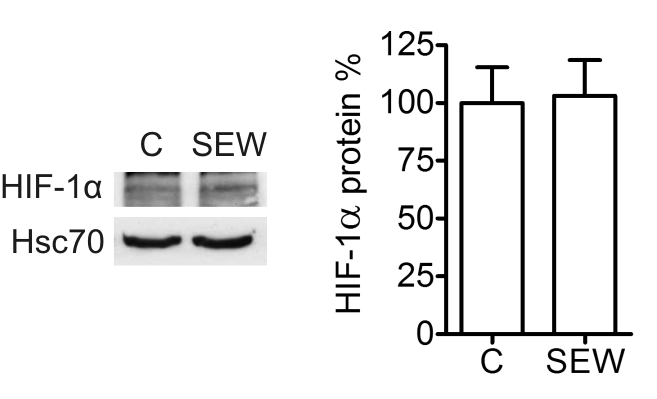

Supplement: Figure S2 — S1P1 activation does not increase HIF-1α expression in ML-1 cells. Cells were treated with 10 µM SEW-2871 (SEW) for 6 h. Result is mean ± SEM, n = 6. (TIF) [file pone.0066189.s002.tif]

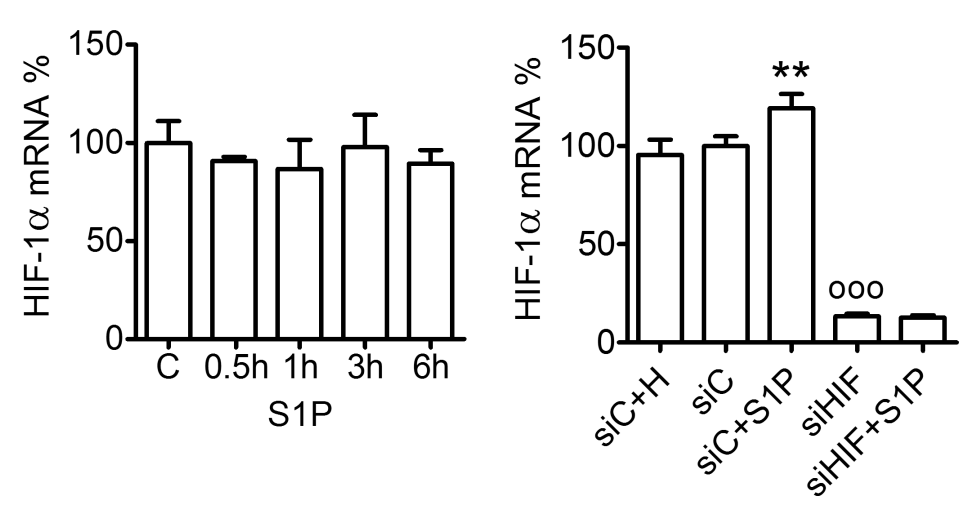

Supplement: Figure S3 — S1P up-regulates HIF-1α mRNA only after long S1P incubation in ML-1 cells. (A) The initial S1P-induced HIF-1α expression is not mediated by increased transcription. Cells were treated with S1P (100 nM) for the indicated times. (B) siRNA against HIF-1α caused an approximately 90% knockdown of HIF-1α mRNA. Cells were transfected with control siRNA (siC) or HIF-1α siRNA (siHIF) and treated with S1P (100 nM) or incubated in hypoxia (1% O2) for 9 h. Results are mean ± SEM, n ≥ 3. **P < 0.01 and ***P < 0.001 indicate statistically significant difference between S1P treatment and vehicle control, oooP < 0.001 indicates significant difference between HIF-1α siRNA and control siRNA. (TIF) [file pone.0066189.s003.tif]

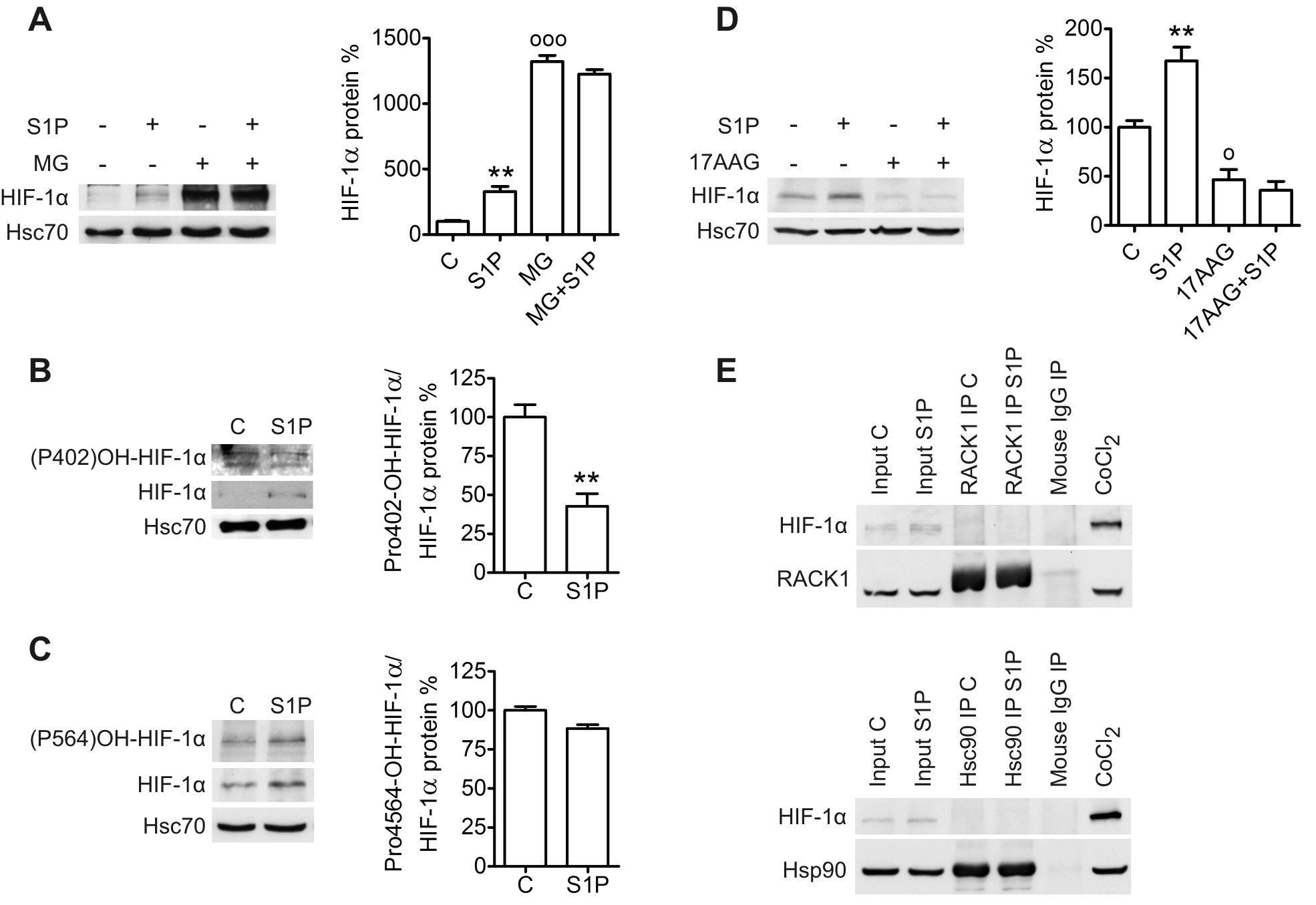

Supplement: Figure S4 — S1P may affect HIF-1α stability. (A) Inhibition of proteasomes strongly elevates the basal HIF-1α protein level and S1P is not able to increase it further. Cells were pre-incubated with MG-132 (MG, 20 µM, 1 h) and stimulated with S1P (100 nM) for 6 h. (B-C) S1P inhibits hydroxylation of HIF-1α on Pro402 but does not inhibit hydroxylation of Pro564. Cells were treated with S1P (100 nM) for 6 h. (D) Inhibition of Hsp90 decreases basal HIF-1α expression and prevents S1P-induced up-regulation of HIF-1α. Cells were pre-incubated with 17-(allylamino)-17-desmethoxygeldanamycin (17-AAG, 2 µM, 16 h) and stimulated with S1P (100 nM) for 6 h. (D) RACK1 and Hsp90 may not bind to HIF-1α in ML-1 cells. Cells were treated with S1P (100 nM) for 6 h. Lysates were immunoprecipitated with a RACK1 or Hsp90 antibody or an IgG control. A lysate of CoCl2-treated cells was used as positive control for HIF-1α. Results are mean ± SEM, n ≥ 3. **P < 0.01 indicates statistically significant difference between S1P treatment and vehicle control, oP < 0.05 and oooP < 0.001 indicate statistically significant difference between inhibitor treatment and vehicle control. (TIF) [file pone.0066189.s004.tif]

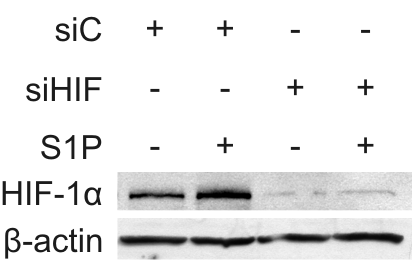

Supplement: Figure S5 — HIF-1α siRNA caused a knockdown of approximately 90% (in the qPCR experiments) and prevented S1P-induced HIF-1α expression. Cells were transfected with control siRNA (siC) or HIF-1α siRNA (siHIF) and treated with S1P (100 nM) for 6 h. (TIF) [file pone.0066189.s005.tif]

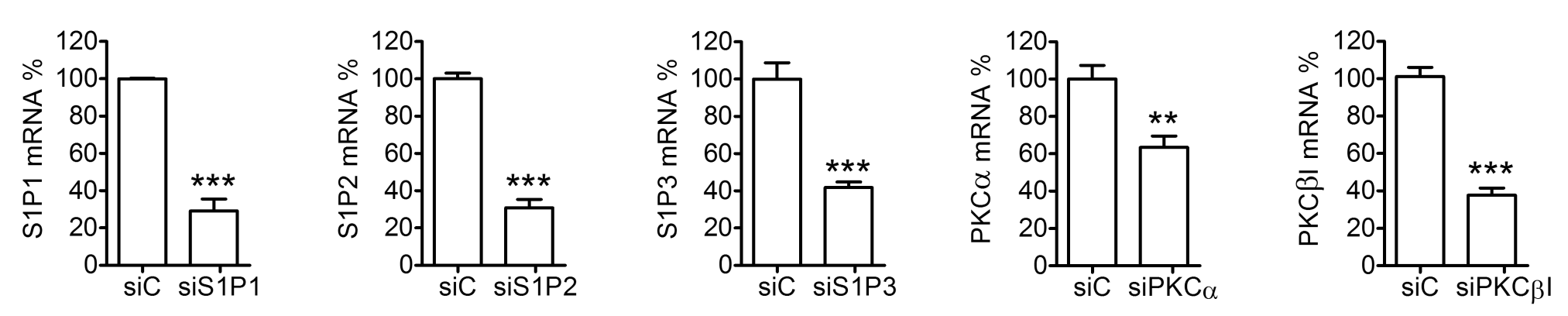

Supplement: Figure S6 — qPCR results showing expression of targeted mRNAs. siRNAs against S1P1, S1P2, S1P3 and PCKβI caused a knockdown of 60–70% and siRNA against PKCα caused a knockdown of approximately 35%. Results are mean ± SEM, n ≥ 5. **P < 0.01 and ***P < 0.001 indicate statistically significant difference between control siRNA and targeting siRNA. (TIF) [file pone.0066189.s006.tif]
