# Supplementary material for: Sphingosine-1-Phosphate as a Regulator of Hypoxia-Induced Factor-1α in Thyroid Follicular Carcinoma Cells
Source: PLoS One. 2013 Jun 18;8(6):e66189. doi: 10.1371/journal.pone.0066189 (PMC3688870; doi:10.1371/journal.pone.0066189)
Supplement: Table S1 — Primer information. (DOC) [file pone.0066189.s007.doc]

**Table S1. Primer information.**

| **Gene** | **Forward primer** | **Reverse primer** | **Probe** |
| --- | --- | --- | --- |
| HIF-1α | 5'-tttttcaagcagtaggaattgga-3' | 5'-gtgatgtagtagctgcatgatcg-3' | UPL #66 |
| VEGF-A | 5'-ccttgctgctctacctccac-3' | 5'-ccacttcgtgatgattctgc-3' | UPL #29 |
| AMF | 5'-cactgcagccagtaatcctct-3' | 5'-gggcatagtggtacaacctgtag-3' | UPL #30 |
| TGFα | 5'-ttgctgccactcagaaacag-3’ | 5'-atctgccacagtccacctg-3' | UPL #63 |
| HPRT | 5'-tgaccttgatttattttgcatacc-3' | 5'-cgagcaagacgttcagtcct-3' | UPL #73 |
| GAPDH | 5'-gttcgacagtcagccgcatc-3' | 5'-ggaatttgccatgggtgga-3' | 5'-accaggcgcccaatacgaccaa-3' |
| S1P_1_ | 5’-gagcactacgcagtcagtcg-3’ | 5’- ttttccttggctggagagg-3’ | UPL #74 |
| S1P_2_ | 5’-ccactcggcaatgtacctgt-3’ | 5-acgcctgccagtagatcg-3' | UPL #61 |
| S1P_3_ | 5’-cttacgacgccaacaagagg-3’ | 5’- aaggcaatgagccagcac-3’ | UPL #69 |
| PKCα | 5’-tcgactgggaaaaactggag-3’ | 5’-ctctgctcctttgccacac-3’ | UPL #83 |
| PKCβI | 5’-agcggtgccatgaatttg-3’ | 5’-tgtggatcttaaacttgtgtttgc-3’ | UPL #2 |
